# Improving childhood vaccination in low-income and middle-income countries

**DOI:** 10.1016/j.ebiom.2023.104617

**Published:** 2023-05-10

**Authors:** eBioMedicine

The goal of World Immunisation Week 2023 is to reach the millions of children worldwide who have not been vaccinated to catch-up on lost progress in essential immunisation. In 2021, approximately 25 million infants had not received important basic vaccines, and the number of unvaccinated children had increased by 5 million since 2019. This global decline in childhood vaccination has been most prevalent in low-income and middle-income countries, in which the burden of infectious diseases is highest. In these countries, infectious diseases are one of the leading causes of child death. Inequalities in socioeconomic status, education, and geographical access continue to impact vaccine effects and dropout rates. Providing novel vaccines to children in low-income and middle-income countries is one way to overcome these issues and substantially reduce the burden of infectious diseases.

Children younger than 6 months have the highest burden of severe disease associated with respiratory syntactical virus (RSV) in some low-income and middle-income countries, as shown in 2023 by Bryan Nyawanda and colleagues in *BMC Medicine*. In *The Lancet*, You Li and colleagues showed via a systematic analysis that there were 3.6 million RSV-associated acute lower respiratory infection hospital admissions and 101,400 RSV-attributable overall deaths worldwide in children aged 0–60 months in 2019. However, no licenced vaccines for RSV exist currently. The only preventive product that is widely available is palivizumab, a monoclonal antibody used to passively immunise clinically vulnerable children and infants against RSV. However, a systematic review published by Rachel Wittenauer and colleagues in *BMC Medicine* in 2023 showed that the cost and requirement of access to a dose every month mean that palivizumab is a less appealing option in low-income and middle-income countries than a vaccine would be. Fortunately, development towards improved immunisation against RSV is ongoing, with multiple vaccine and antibody products for RSV prevention currently being tested. One promising area of development is immunisation of the birthing parent (also known as maternal vaccination). The PREPARE trial, the first phase 3 trial of a vaccine for RSV in birthing parents published by Shabir Madhi and colleagues in *The New England Journal of Medicine* in 2020, randomly assigned 4636 pregnant people with an expected delivery date near the start of the RSV season to receive a single intramuscular dose of either RSV vaccine or placebo. Conducted in multiple low-income, middle-income, and high-income countries, the primary outcome was RSV-associated, medically significant lower respiratory tract infection in the infants up to age 90 days. Although the vaccine did not meet its primary outcome, it was established to be safe and provided a proof-of-concept of maternal vaccination against RSV (medically significant lower respiratory tract infection was 1.5% in the vaccine group and 2.4% in the placebo group). Furthermore, the PREPARE trial showed long-term efficacy of maternal vaccination against other conditions, such as all-cause infant pneumonia, showing its importance in low-income and middle-income countries where vaccination coverage is most scarce. Even more common conditions, such as asthma, can be prevented via RSV vaccination of the birthing parent according to a modelling study published by Justin Ortiz and colleagues in the *Journal of Allergy and Clinical Immunology* in 2023, which will further reduce the overall disease burden. However, the mechanisms of cross protection are unknown and worthy of further investigation.

Fortunately, the overall disease burden has already been somewhat reduced by developments in vaccination and immunisation against other infectious diseases. For example, almost every child had been infected by a rotavirus before age 5 years and rotaviruses caused approximately 500 000 deaths and 2 million hospitalisations worldwide each year before the introduction of a preventive vaccine in 2006. This preventive vaccine also led to a global reduction in the incidence of rotavirus-associated gastroenteritis. In countries that have not yet introduced rotavirus vaccination, however, the disease burden and corresponding economic costs remain high. A modelling study published in *The Lancet Global Health* in 2021 evaluated the cost–benefit ratio of a rotavirus vaccine for children younger than 1 year in the Central African Republic, Chad, Comoros, Equatorial Guinea, Gabon, Guinea, Somalia, and South Sudan. They reported that the benefit of vaccine introduction outweighed the costs in all countries. A survey of the genetic diversity of circulating strains of rotavirus A in Gabon, published in *eBioMedicine* in 2021, detailed high levels of antigenic variability, which further highlights the need to urgently introduce national rotavirus A vaccination programmes. An additional concern is incomplete protection against infection with the current generation of vaccines. RotaTeq and Rotarix, two of the four oral rotavirus vaccines prequalified for global use by WHO, had excellent efficacy in clinical trials in high-income countries, but vaccine efficacy was substantially diminished in trials conducted in low-income and middle-income countries, as reviewed by Benjamin Lee in *Human Vaccines and Immunotherapics* in 2021. This underperformance, however, is multifactorial, and might not be due to the vaccines themselves. For example, limitations in methods for measuring immune response and protection and not accounting for decreased natural susceptibility to rotavirus infections were both shown to decrease reported estimates of vaccine efficacy. Improving the methods of assessment of vaccine efficacy could lead to higher-quality research, results, and reduction of overall disease burden. As stated by Daniel Velasquez-Portocarrero and colleagues in *The Lancet Infectious Diseases*, additional vaccination strategies should be evaluated to overcome the suboptimal performance of current oral rotavirus vaccines in low-income and middle-income countries.

Another infectious disease against which vaccination and immunisation would reduce the overall disease burden in low-income and middle-income countries is tuberculosis. Despite a reduction in tuberculosis cases during the COVID-19 pandemic, the incidence has begun to increase once again; 6.4 million new cases were reported worldwide in 2021. Tuberculosis is expected to soon return to being the deadliest infectious disease globally and, without treatment, the mortality rate from tuberculosis is high. Two-thirds of new tuberculosis cases occur in eight low-income and middle-income countries (ie, India, China, Indonesia, Philippines, Pakistan, Nigeria, Bangladesh, and South Africa) and although some candidate vaccines have potentially strong protection against tuberculosis, they require refrigerated storage, which is substantially more difficult to provide in low-income and middle-income settings than in high-income settings. In a randomised, double-blind, phase 1 clinical trial of healthy adults aged 18–48 years published by Zachary Sagawa and colleagues in *Nature Communications* in 2022, a thermostable vaccine candidate against tuberculosis was safe and well tolerated, and elicited robust immune responses. However, as this trial was conducted in adults in the USA, the extent to which these findings can be generalised to children in low-income and middle-income countries is questionable. Unfortunately, an analysis of notification data published in *The Lancet Global Health* by Lasith Ranasinghe and colleagues in 2022 found that COVID-19 has substantially affected childhood tuberculosis services, indicating that even if a vaccine was shown to be effective in children in low-income and middle-income countries, real-life administration to all children who need it would be difficult. Furthermore, according to Hazel Dockrell and Helen McShane in their 2022 *eBioMedicine* personal view, research and development of new tuberculosis vaccines has been slow because of poor funding, another setback to vaccine provision that disproportionately affects low-income and middle-income settings. However, a systematic review and individual participant data meta-analysis published in *The Lancet Global Health* by Leonardo Martinez and colleagues in 2022 showed that the only licenced vaccine against *Mycobacterium tuberculosis* currently, the BCG vaccine, protected infants and children younger than 5 years against all tuberculosis when results were stratified by age. Moreover, WHO announced in January, 2023, their plan to establish a new Tuberculosis Vaccine Accelerator Council. This council hopes to identify and overcome current issues in tuberculosis vaccine development. These commitments and developments are promising regarding increasing vaccination and reducing the burden of infectious diseases in low-income and middle-income countries.

Although ongoing action and research make the future of immunisation look increasingly favourable, there is still a substantial deficit in the provision and administration of vaccines in low-income and middle-income countries. Global stakeholders should improve their collaboration with and support of researchers and clinicians in low-income and middle-income countries to establish safe and effective vaccines for children to reduce the burden of infectious diseases. Currently, African researchers are looking at ways to produce local vaccines, and the rest of the world should support this endeavour with funding and knowledge exchange. *eBioMedicine* welcomes all research and development in this field and encourages submissions from individuals from low-income and middle-income countries.

